# The MAP Kinase PvMK1 Regulates Hyphal Development, Autophagy, and Pathogenesis in the Bayberry Twig Blight Fungus *Pestalotiopsis versicolor*

**DOI:** 10.3390/jof9060606

**Published:** 2023-05-24

**Authors:** Xiujuan Yan, Shuwen Zhang, Zheping Yu, Li Sun, Muhammad Aamir Sohail, Zihong Ye, Lei Zhou, Xingjiang Qi

**Affiliations:** 1 Zhejiang Provincial Key Laboratory of Biometrology and Inspection & Quarantine, College of Life Sciences, China Jiliang University, Hangzhou 310018, China; s20090710062@cjlu.edu.cn (X.Y.); zhye@cjlu.edu.cn (Z.Y.); 2 State Key Laboratory for Managing Biotic and Chemical Threats to Quality and Safety of Agro-Products, Institute of Horticulture, Zhejiang Academy of Agricultural Sciences, Hangzhou 310021, China; hizhangshuwen@163.com (S.Z.); yuzp@zaas.ac.cn (Z.Y.); sunl@zaas.ac.cn (L.S.); 3 Key Laboratory of Detection for Pesticide Residues and Control of Zhejiang, Institute of Agro-Product Safety and Nutrition, Zhejiang Academy of Agricultural Sciences, Hangzhou 310021, China; amirsohail306@gmail.com; 4 Biotechnology Research Institute, Xianghu Laboratory, Hangzhou 310021, China

**Keywords:** *Pestalotiopsis versicolor*, MAP kinase, stress response, autophagy, pathogenesis

## Abstract

Bayberry twig blight caused by the ascomycete fungus *Pestalotiopsis versicolor* is a devastating disease threatening worldwide bayberry production. However, the molecular basis underlying the pathogenesis of *P. versicolor* is largely unknown. Here, we identified and functionally characterized the MAP kinase PvMk1 in *P. versicolor* through genetic and cellular biochemical approaches. Our analysis reveals a central role of PvMk1 in regulating *P. versicolor* virulence on bayberry. We demonstrate that PvMk1 is involved in hyphal development, conidiation, melanin biosynthesis, and cell wall stress responses. Notably, PvMk1 regulates *P. versicolor* autophagy and is essential for hyphal growth under nitrogen-depleting conditions. These findings suggest the multifaceted role of PvMk1 in regulating *P. versicolor* development and virulence. More remarkably, this evidence of virulence-involved cellular processes regulated by PvMk1 has paved a fundamental way for further understanding the impact of *P. versicolor* pathogenesis on bayberry.

## 1. Introduction

Bayberry produced by the *Myrica rubra* tree is a subtropical fruit with a unique sweet and sour taste native to southern China, which is commercially cultivated in many areas of China, including Zhejiang, Guizhou, Guangxi and Yunnan provinces, and has become an important economic pillar for local mountain farmers [1,2,3]. Besides its economic significance, recent studies have shown that bayberry also has nutritional and medicinal value; indeed, its extracts exhibit anti-cancer and anti-oxidant activity, making this a promising fruit regarding its potential to be industrially processed for high-value-added food products [4,5,6,7,8]. However, in recent years, bayberry production has been threatened by a devastating disease called twig blight, which is caused by the ascomycete fungi *Pestalotiopsis versicolor* and *P. microspore* [9]. This disease was first discovered in Zhejiang in 2004, and has now been widely spread in bayberry planting areas, becoming a major disease endangering bayberry production. The disease symptoms firstly appear in young twigs and the associated leaves, causing the infected twigs and leaves to turn brown, scorched, and eventually the whole tree to defoliate after one to two months. The whole crop of severely infected bayberry trees will then die, especially those aged 10 to 15 years, leading to huge yield losses in bayberry production [10]. In addition to twig blight disease on *Myrica rubra* trees, *Pestalotiopsis* pathogens also cause leaf spot diseases on many other economically important fruit and medicinal plants, such as banana, citrus, green tea, and ginseng [11,12,13,14]. Although more and more diseased plant species have been reported for *Pestalotiopsis* fungi, the molecular mechanisms underlying their pathogenesis are largely unknown.

The mitogen-activated protein (MAP) kinase cascades are well-conserved signaling pathways present in eukaryotic organisms, from yeast to plants and animals, and are responsible for sensing environmental signals and regulating self-development [15]. In the budding yeast Saccharomyces cerevisiae, there are five MAPK pathways, namely Fus3, Kss1, Slt2, Hog1, and Smk1, involved in its mating, invasive growth, cell wall integrity, osmoregulation, and ascospore formation, respectively [16]. In contrast, in plant pathogenic fungi, three MAPK pathways are commonly identified. One, which involves the yeast Fus3/Kss1 orthologs, regulates hyphae development and the formation of the infection structure. In the rice blast fungus *Magnaporthe oryzae*, the pathogenicity MAP kinase 1 (Pmk1) is essential for appressorium formation and invasive growth [17]. These Fus3/Kss1-type MAPKs have been extensively studied in more than 20 plant pathogenic fungi and have shown to be essential for fungal pathogenicity by modulating the attachment and sensing of host plant signals [18]. The second, which involves the yeast Slt2 orthologs, regulates cell wall integrity (CWI) signaling in response to external or internal cell wall stress conditions. These Slt2-type MAPKs also have conserved roles in controlling fungal pathogenicity and are also involved in regulating other developmental processes in plant pathogenic fungi [19]. For example, in *M. oryzae*, the mps1 (a yeast Slt2 ortholog) mutant is lost in virulence and it has severe defects regarding its aerial hyphal growth and conidiation, suggesting that MAPKs play multiple roles in plant pathogenic fungi [20]. The third is the osmoregulation pathway, which is governed by the Hog1 orthologs that are required for fungi to respond to high-osmotic stresses [21]. These Hog1-type MAPKs in plant pathogenic fungi exhibit a species-specific role in fungal pathogenesis. Whereas the Hog1 MAPK in the wilt fungus *Verticillium dahliae* is essential for plant infection, its homolog in *M. oryzae*, the Osm1 MAPK, is dispensable for rice infection [22,23,24,25]. Hog1-type MAPKs are also involved in the regulation of other biological processes, such as melanin biosynthesis and fungicide resistance [25,26,27,28]. Recent increasing evidence has shown that MAPK pathways cross talk regarding the regulation of the development and pathogenicity of plant fungal pathogens. For example, in the vascular wilt fungus *Fusarium oxysporum*, both the Fus3/Kss1-type MAPK Fmk1 and the Slt2-type MAPK Mpk1 are involved in the regulation of fungal responses to cell wall and heat stresses [29,30]. These findings provide evidence that the yeast MAPK orthologs play conserved roles in regulating the pathogenicity of plant pathogenic fungi and have undergone functional differentiation in response to changing environmental conditions.

Autophagy is an evolutionarily conserved process in eukaryotes used to maintain survival under starvation conditions via the degradation of intracellular damaged proteins and organelles for nutrient recycling [31]. Besides its medical significance in regulating many human diseases, this process also plays an important role in controlling fungal growth and development [32]. The molecular mechanisms underlying this self-degradation process have been extensively studied in the budding yeast *S. cerevisiae*, where a total of 31 autophagy-related genes (ATGs) have been identified and functionally characterized [33]. These findings have provided evidence that autophagy can help organisms to cope with nutrient-depleting conditions to survive for a longer time. Many recent studies have demonstrated that autophagy is pivotal for the pathogenicity in most fungal plant pathogens [34]. In the rice blast fungus *M. oryzae*, the deletion of the autophagy gene *MoATG8* abolished the capacity of the fungus to cause disease on rice due to its inability to form the infection structure appressorium and to penetrate the rice cuticle [35,36]. This critical role of autophagy in fungal infection has also been illustrated in many other plant pathogens, such as the wheat blight fungus *Fusarium graminerum*, the tomato wilt fungus *F. oxysporum*, the gray mold fungus *B. cinerea*, and the anthracnose disease-causing Colletotrichum pathogens [37,38,39,40,41]. In these fungi, autophagy is essential for their formation of infection structures, suggesting the conserved role of autophagy in fungal pathogenicity. A common mechanism underlying its virulence-regulating role is that autophagy can provide nutrient materials for fungal invasive growth and infection structure differentiation under starvation conditions on the plant surface. This starvation signal is possibly perceived by the Fus3/Kss1-type MAPK signaling pathway in *M. oryzae*, as demonstrated recently by Míriam et al., in which the MAP kinase Pmk1 directly and specifically activated its downstream transcription factor MoHox7 in order to initiate autophagy at the beginning of plant infection [42].

In this study, we set out to understand the molecular basis underlying *P. versicolor* pathogenesis on bayberry. We demonstrate that the MAP kinase PvMk1 plays important roles in *P. versicolor* hyphal growth, conidiation, virulence, and autophagy under nutrient-depleting conditions. Moreover, PvMk1 is involved in the stress response of *P. versicolor* to cell-wall-damaging drugs, suggesting a crosstalk between the PvMk1 signaling cascades and the cell wall integrity pathway in *P. versicolor*.

## 2. Materials and Methods

### 2.1. Fungal Strains and Culture Conditions

The *P. versicolor* strain XJ27 served as the wild-type strain [9]. XJ27 and its derivatives were grown on potato dextrose agar (PDA, 200 g of potato, 20 g of glucose, 15 g of agar and 1 L of H_2_O) or complete medium (CM, 10 g of glucose, 2 g of peptone, 1 g of yeast extract, 1 g of casamino acids, 15 g of agar, 1 mL of vitamin solution [0.01 g of biotin, 0.01 g of pyridoxine, 0.01 g of thiamine, 0.01 g of riboflavin, 0.01 g of p-aminobenzoic acid and 0.01 g of nicotinic acid in 100 mL of distilled water], 1 mL of trace elements [2.2 g of ZnSO_4_•7H_2_O, 1.1 g of H_3_BO_3_, 0.5 g of MnCl_2_•4H_2_O, 0.5 g of FeSO_4_•7H_2_O, 0.17 g of CoCl_2_•6H_2_O, 0.16 g of CuSO_4_•5H_2_O, 0.15 g of Na_2_MnO_4_•2H_2_O and 5 g of Na_4_EDTA in 100 mL of distilled water, pH 5.8], 50 mL of 20× nitrate salts [120 g of NaNO_3_, 10.4 g of KCl, 10.4 g of MgSO_4_•7H_2_O, 30.4 g of KH_2_PO_4_ in 1 L of distilled water] in 1 L of distilled water) or minimal medium (MM, 10 g of D-glucose, 1 mL of trace elements, 50 mL of 20× nitrate salts, 0.01 g of thiamine and 15 g of agar in 1 L of distilled water) for mycelial growth tests and conidiation assays.

### 2.2. Genome Sequencing and Annotation

Genome sequencing used the nanopore ONT platform. Nanopore reads were performed using Next Denovo v2.5.0 software (https://github.com/Nextomics/NextDenovo, accessed on 16 May 2021). The default parameters were spliced. The contig sequence obtained was corrected using pilon software (version 1.23) and second-generation sequencing data [43]. EDTA v2.1.0 software (https://github.com/oushujun/EDTA, accessed on 19 May 2021) was used for the identification of the genome repeat sequence [44]. The repetitive sequence was used for gene annotation by employing a soft-masked genome, and the software used was BRAKER2 (https://github.com/Gaius-Augustus/BRAKER, accessed on 23 May 2021). During annotation, RNA-Seq data were used to train the gene prediction software embedded in braker2 [45]. BUSCO v3.0.2 software was used to evaluate the completeness of gene prediction [46]. AHRD v3.3.3 software (https://github.com/groupschoof/AHRD, accessed on 26 May 2021) was used for the functional annotation of the predicted genes.

### 2.3. Targeted Gene Deletion and Complementation

The vector was constructed using a one-step recombination cloning method, as shown in the schematic diagram in Figure S1A. Briefly, the ~1.0 kb upstream fragment and the ~1.0 kb downstream fragment flanking the *PvMK1* gene were amplified using the genomic DNA of *P. versicolor* and the primers Y0063F/R and Y0064F/R, respectively. A ~1.4 kb fragment of the hygromycin B resistance gene cassette (HPH) was amplified using the pBHt2 vector and ligated into the EcoRI site of the pDHt2 vector to make pCH [47,48]. The upstream and downstream sequences were then ligated to both sides of HPH to produce the final vector pCH-PvMK1 (Figure S1A). For target gene deletion, the pCH-PvMK1 vector was transformed into the *P. versicolor* strain XJ27 using an Agrobacterium tumefaciens-mediated transformation (ATMT) method, as previously described [48]. Putative transformants were selected on PDA plates supplemented with hygromycin B at a concentration of 50 μg/mL. Hygromycin-resistant transformants were further detected using southern blot analysis and the HPH fragment as a probe to confirm the single insertion of the T-DNA region in the vector pCH-PvMK1. Then, combined primers Y0044F/Z0074R and Z0074F/Y0044R were used to confirm the in-situ replacement of the *PvMK1* gene with the HPH fragment (Figure S1B–D). For mutant complementation, a 3.6 kb fragment containing the *PvMK1* CDS region and its upstream promoter and downstream terminator sequences were amplified using primers Z0660F/R and subsequently cloned into the pCOM vector to generate pCOM-PvMK1 [48]. The pCOM-PvMK1 vector was transformed into the *PvMK1* gene deletion mutant using the ATMT method, which was selected by geneticin G418 resistance at a concentration of 50 μg/mL. The gene deletion and complementation transformants were further confirmed via RT-PCR using primers Y0154F/R to detect the loss and normal expression of the *PvMK1* gene, respectively (Figure S1E). All primers used were listed in Supplementary Table S1.

### 2.4. Phenotypic Analysis

For assays of vegetative growth and conidiation, the wild-type XJ27, Δ*Pvmk1* mutant, and Δ*Pvmk1-C* were cultured on PDA, CM, and MM plates containing different nutritional ingredients at 25 °C for 6 d. The colony area was measured using Image J v1.8.0 (https://imagej.en.softonic.com/, accessed on 16 November 2022). The number of conidia was measured on PDA plates cultured for 15 d. Briefly the plate was washed with 10 mL of sterile water to obtain a spore suspension. A hemocytometer was used to count the spores under a microscope and calculate the spore concentration. To test the response to osmotic stress, the wild-type XJ27, Δ*Pvmk1* mutant and Δ*Pvmk1-C* were cultured on PDA plates supplemented with 2 M of sorbitol or 0.5 M of NaCl. For stress assays of the cell wall integrity, XJ27, Δ*Pvmk1* and Δ*Pvmk1-C* strains were inoculated on PDA plates containing 100 μg/mL of CR or 300 μg/mL of CFW. To test their sensitivity to oxidative stress, each strain was grown on PDA plates containing 20 mM ofH_2_O_2_. Photographs were taken and the colony area were measured at 6 d post-inoculation. All treatments were repeated three times.

### 2.5. Virulence Assays

For the virulence assay, mycelial plugs of the wild-type XJ27, Δ*Pvmk1* mutant and Δ*Pvmk1-C* were inoculated on the wounded leaves of *Myrica rubra* (Cultivar ‘Zaojia’) under 28 °C with a 12 h light/12 h dark photoperiod for 3 d. For each strain, 10 replicates of the inoculation experiments were performed. The lesion area on the leaves was determined using Image J. To observe the infection structures and penetration sites, conidia of XJ27, Δ*Pvmk1*, and Δ*Pvmk1-C* strains were adjusted to 10^4^ spore/mL, 10 μL of which was taken to inoculate the inner epidermis of an onion. The infectious hyphae development and penetration sites were observed under microscopy at 24 h post-inoculation.

### 2.6. Quantitative Real-Time PCR (qRT-PCR)

The total RNA was extracted from the mycelia of the wild-type XJ27 and Δ*Pvmk1* mutant harvested from the liquid CM that was inoculated and incubated at 25 °C with constant shaking at 180 rpm for 3 d, using a RNA simple Total RNA Kit (TIANGEN, Beijing, China). Reverse transcription was used to generate cDNA using a reverse transcription kit (TIANGEN, Beijing, China). Quantitative PCR was conducted using a SYBR kit (Vazyme, Nanjing, China) with specific primers (Table S1). *P. versicolor* β-actin (Table S1) was used as the endogenous reference gene, and the expression levels were calculated using the 2^−ΔΔCt^ method, as previously reported [49]. All qRT-PCR assays were repeated three times for each sample.

### 2.7. Western Blot and MAPK Phosphorylation Assays

The fresh mycelia (100 mg) of each strain were finely ground and suspended in 500 μL of extraction buffer (50 mM of Tris-HCl, pH7.5, 100 mM of NaCl, 0.5 mM of EDTA, 1% Triton X-100, 1 mM of PMSF) and a 1× protease inhibitor cocktail (TargetMol, Shanghai, China). After homogenization with a vortex shaker, the lysate was centrifuged at 10,000× *g* in a high-speed freezing centrifuge for 30 min at 4 °C. The resulting proteins were separated on 12% denatured polyacrylamide gel (SDS-PAGE) and transferred to an Immobilon-P transfer membrane (Millipore, Carrigtwohill, Ireland) with a Bio-Rad electroblotting apparatus. The phosphorylation level of PvSlt1 and PvSlt2 was detected using an anti-phospho-p44/42 MAPK antibody (Cell Signaling Technology, Boston, MA, USA). The total protein of PvSlt1 and PvSlt2 was detected using an p44/42 MAPK antibody (Cell Signaling Technology, Boston, MA, USA). Incubation with a secondary antibody and chemiluminescent detection was performed as described previously [50]. In addition, in order to detect phosphorylated PvHog1 under hypertonic conditions, total proteins were extracted at different time points from the hyphae grown in the liquid CM supplemented with 0.5 M of NaCl. We used an anti-MAPK14 (T180/Y182) antibody (Cell Signalling Technology, Boston, MA, USA) and an anti-MAPK14 antibody (Cell Signalling Technology, Boston, MA, USA) to detect nonphosphorylated and phosphorylated PvHog1 protein levels. Three independent experiments were conducted [50].

### 2.8. Autophagy Assays

After culturing the wild-type strain XJ27 and mutant strain Δ*Pvmk1* in CM medium for 3 d, mycelia were taken from the edge of the plate and suspended in 500 μL of ddH_2_O, and 100 μL of hyphal suspension was taken, transferred into CM liquid medium and shook at 200 rpm for 72 h at 25 °C. After the culture, the fungal suspension was centrifuged at 6000 rpm for 5 min, and the supernatant was discarded. Then, the pellet was washed twice with ddH_2_O, transferred to MM liquid medium (containing 2 mmol/L PMSF), and incubated in a shaker at 25 °C for 4 h with 200 rpm. After 4 h, the mycelium was collected via centrifugation and shifted into a 2.5% glutaraldehyde solution at 4 °C for overnight fixation. The sample was then treated according to the following steps: rinse the sample with phosphoric acid buffer (0.1 M PH 7.0) 3 times, 10 min each time, fix the sample with 2% osmic acid for 1–2 h, rinse the sample with phosphoric acid buffer 3 times, 10 min each time, perform 50%, 70%, 80%, 90% and 95% ethanol gradient dehydration for 10 min each time, and finally perform anhydrous ethanol dehydration twice. After 20 min each time, finally use acetone to dehydrate the sample for 20 min, and put the sample into the mixture of Spurr and acetone in the ratio of 1:1 for 1 h, and then into the mixture of Spurr and acetone in the ratio of 3:1 for 3 h. Then, change to a new tube and use a 100% Spurr embedding agent to permeate overnight. Add a 100% Spurr embedding agent into a 500 mL centrifuge tube, place the sample at the cutting position at the bottom of the tube, polymerize it at 70 °C for 24 h, slice it in an ultrathin microtome, dye it using a lead citrate solution and uranyl acetate 50% ethanol-saturated solution for 15 min, respectively, and observe the sample under the Hitachi H7650 transmission electron microscope [51].

### 2.9. Statistical Analysis

Each experiment was performed with at least three replicated measurements. The values of the results were represented as the mean ± standard deviation (SD). The significant differences between treatments were statistically determined via one-way analysis of variance (ANOVA) comparison using SPSS (version 26.0, IBM, Almond, New York, NY, USA), which was followed by a *t*-test to determine whether the ANOVA analysis was significant at *p* < 0.05 or *p* < 0.01.

## 3. Results

### 3.1. Genome Sequencing of P. versicolor Strain XJ27 and Functional Annotation

A high-quality genome will be generally very helpful for the study of functional genomics in fungi [52]. However, currently there is no available genome assembly of *P. versicolor* fungus deposited in the NCBI database. Therefore, we first set out to sequence the whole genome of our *P. versicolor* XJ27 strain, which showed high virulence to bayberry trees. *P. versicolor* genomic DNA was extracted and sequenced on the Oxford Nanopore Technologies (ONT) platform. Raw reads were generated and assembled into the final genome of 50,743,142 bp containing 8 scaffolds with N50 values of 7,257,579 bp and a 52.93% GC content. Using the homology-based gene predictor of Exonerate v2.4.7 [53], 16,445 protein-encoding genes were identified in the genome, with an average gene length of 665 bp. For the prediction of secreted proteins, combination analyses using Signal P, Target P, TMHMM were performed; these identified 1884 putative secreted proteins with N-terminal signal peptides and no transmembrane regions [54,55,56]. To evaluate the genome completeness of the assembly and annotation, BUSCO v4.0.0 [46] was performed using 1315 Ascomycota single-copy Benchmarking Universal Single-Copy Orthologs (BUSCOs), which showed that 99.8% of the gene sets in the current genome assembly consisted of whole, single-copy BUSCOs (Table 1). These results indicated that the *P. versicolor* is a haploid fungus and that the genome here was well assembled with a high completeness.

### 3.2. Identification of PvMk1 and Targeted Gene Deletion

**Figure 1:**
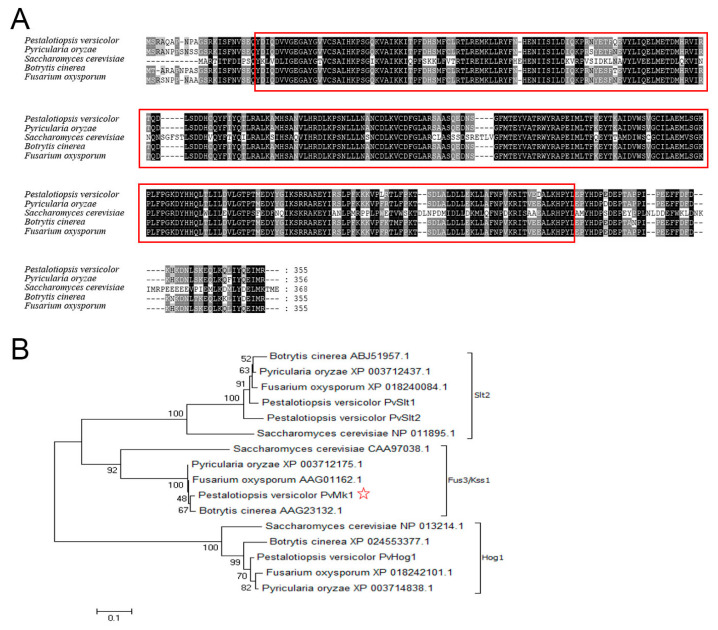
Characterization of the PvMk1 protein in *P. versicolor*. (**A**) Alignment analysis of the amino acid sequences of PvMk1 and its homologs from other fungi using the DNAMAN program. Red circled represents the typical S-TKc domain sequences. Kss1: *S. cerevisiae* (CAA97038.1); PMK1: *M. oryzae* (XP_003712175.1); BMP1: *B. cinerea* (AAG23132.1); FMK1: *F. oxysporum* (AAG01162.1); (**B**) Phylogenetic analysis of four MAPKs in *P. versicolor* and their homologs from other fungi. Red star marked is the PvMk1 protein in this study. The amino acid sequences were analyzed using MEGA 7.0 and an unrooted neighbor-joining algorithm. Bootstrap values were calculated from 1000 bootstrap replicates. Only bootstrap support values >50% are shown.

To identify the MAP kinase-encoding genes in the *P. versicolor* genome, the amino acid sequences of five MAP kinases, namely Fus3, Kss1, Slt2, Hog1, and Smk1 in the model fungus *Saccharomyces cerevisiae*, were used as queries to search the annotated proteome of *P. versicolor* via the BLASTP algorithm. Four proteins, including one Fus3 homolog PvMk1, two Slt2 homologs PvSlt1 and PvSlt2, and one Hog1 homolog PvHog1, were identified, exhibiting 59.43%, 68.87%, 66.12%, and 83.28% sequence identity, respectively. The *PvMK1* gene has a total length of 1247 bp, which contains an open reading frame of 1065 bp, encodes 355 amino acids, and contains a typical S-TKc domain (Figure 1A). Phylogenetic analysis shows that these four MAPKs identified in *P. versicolor* were classed into three major MAPK families, representing the three typical MAPK pathways in filamentous fungi (Figure 1B).

To facilitate investigation of the physiological role of PvMk1 in *P. versicolor*, we first established an Agrobacterium-mediated transformation method for targeted gene deletion in the *P. versicolor* genome based on homologous recombination [48]. The sensitivity of the *P. versicolor* strain XJ27 to hygromycin B and geneticin was evaluated by culturing the fungus on PDA medium supplemented with different concentrations of hygromycin B and G418 geneticin. The growth of *P. versicolor* was almost completely inhibited on medium containing 50 μg/mL of hygromycin B or 50 μg/mL of G418 geneticin. Thus, we used 50 μg/mL of Hygromycin B or G418 geneticin for *P. versicolor* transformants selection.

A gene knockout vector pDH-PvMK1 was constructed by inserting two flanking sequences of *PvMK1* into both sides of the hygromycin B resistance gene in the pDHt2 vector, which was transformed into the wild-type strain XJ27 as previously described (Figure S1A) [48]. After hygromycin B selection and PCR screening, three deletion mutants with similar phenotypes were obtained. We selected one of the mutants named Δ*Pvmk1* for further analyses. To confirm that the phenotypic differences observed in the gene deletion mutants were associated with the gene replacement event, a wild-type *PvMK1* gene under the control of its native promoter and terminator was introduced into the gene deletion mutant Δ*Pvmk1* using the pCOM-PvMK1 vector. One geneticin-resistant transformant was randomly selected for RT-PCR analysis and it was confirmed that the *PvMK1* gene was normally expressed in the complemented strain, which was designated as Δ*Pvmk1-C* (Figure S1E).

### 3.3. PvMk1 Is Required for Hyphal Development and Conidiation

**Figure 2:**
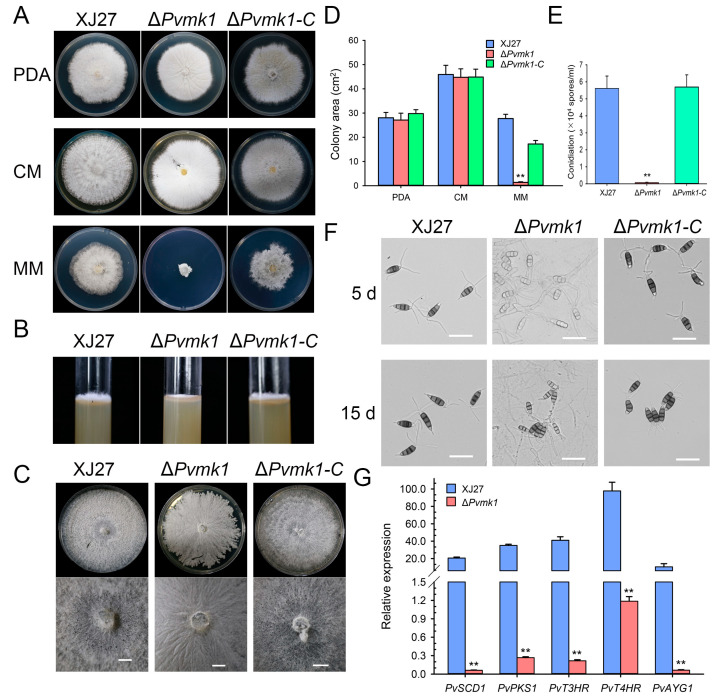
Loss of *PvMK1* gene impairs aerial hyphal growth and spore development. (**A**) The colony morphology of the mutant Δ*Pvmk1*. The wild-type XJ27, the mutant Δ*Pvmk1* and complemented transformant Δ*Pvmk1-C* were inoculated on PDA, CM and MM media at 25 °C in the dark for 6 d. (**B**) Observation of the aerial hyphal development of indicated strains grown on CM medium in test tubes. (**C**) Observation of colony pigmentation of different strains grown on MM medium for 5 d and 15 d. Bar = 1 mm. (**D**) Values of colony area of indicated strains cultured on PDA, CM and MM media at 25 °C in the dark for 6 d. (**E**) Number of conidia produced by indicated strains in 15 d old PDA cultures. (**F**) Conidial pigmentation of XJ27, Δ*Pvmk1* and Δ*Pvmk1-C* were cultured on MM medium for 5 and 15 d. Bar = 25 μm. (**G**) The relative expression of the melanin biosynthesis genes PvPKS1, PvSCD1, PvT3HR, PvT4HR and PvAYG1 in XJ27 and Δ*Pvmk1* cultured on PDA for 10 d. The expression level of β-tubulin gene in *P. versicolor* was used to normalize different samples. Bars represent means and standard deviations (three replications). Asterisks indicate statistical differences from the wild-type XJ27 (** *p* < 0.01). Each experiment was performed with three replications. Asterisks indicate statistical differences from the wild-type XJ27 (** *p* < 0.01). The data were analyzed via a one-way ANOVA followed by Duncan’s test using GraphPad Prism (version 8.0).

To explore the role of PvMk1 in fungal growth and the development of *P. versicolor*, the differences in colony morphology, vegetative growth, and conidiation among the wild-type strain XJ27, the Δ*Pvmk1* mutant, and the complemented strain Δ*Pvmk1-C* were characterized by culturing independently on PDA, CM, and MM media for 5 d. On the PDA and CM plates, there was no significant difference in the colony diameter of the Δ*Pvmk1* mutants compared with the wild-type XJ27 and the complemented strain Δ*Pvmk1-C*. The main difference is that the Δ*Pvmk1* mutant colony surface turned white and the aerial hyphae development was significantly decreased. Interestingly, on MM medium, the Δ*Pvmk1* mutants were basically unable to grow (Figure 2A,D). The growth defect of Δ*Pvmk1* regarding its aerial hyphae formation was further confirmed by the phenotype of these three strains grown on PDA medium in the test tubes (Figure 2B). When the incubation time was extended to 15 d, the Δ*Pvmk1* mutant had some growth on MM medium, but compared to wild-type and complemented strains, the aerial hypha development of the mutant was severely impaired (Figure 2C). These results indicate that PvMk1 is implicated in the regulation of aerial hyphal development in *P. versicolor*.

Next, we examined the conidiation of the Δ*Pvmk1* mutants. Quantitative analysis showed that conidiation in the Δ*Pvmk1* mutant was nearly abolished, whereas the wild-type and complemented strain Δ*Pvmk1-C* produced normal and comparable amounts of conidia, suggesting that PvMk1 plays a key role in *P. versicolor* conidium production (Figure 2E).

Further observation revealed that the conidia produced by the Δ*Pvmk1* mutants were less pigmented than the wild-type and complemented strains, indicating a defect of melanin biosynthesis caused by *PvMK1* gene deletion (Figure 2F). According to the well-studied melanin biosynthesis genes in *M. oryzae* and *V. dahliae*, we identified five homolog genes in the *P. versicolor* genome, which were designated as *PvPKS1*, *PvSCD1*, *PvT3HR*, *PvT4HR*, and *PvAYG1* [57,58]. We then performed qRT-PCR to determine the changes in the expression levels of these melanin biosynthesis genes in the Δ*Pvmk1* mutants (Figure 2G). The results showed that all these five melanin biosynthesis genes were significantly reduced in their expression levels in the Δ*Pvmk1* mutants compared to the wild-type strain XJ27 (gene sequences and primers used for qRT-PCR are listed in Supplementary Tables S1 and S2). These results suggest that PvMk1 regulates melanin biosynthesis in *P. versicolor*.

### 3.4. PvMk1 Regulates Nitrogen Stress Responses Required for P. versicolor Hyphal Development

**Figure 3:**
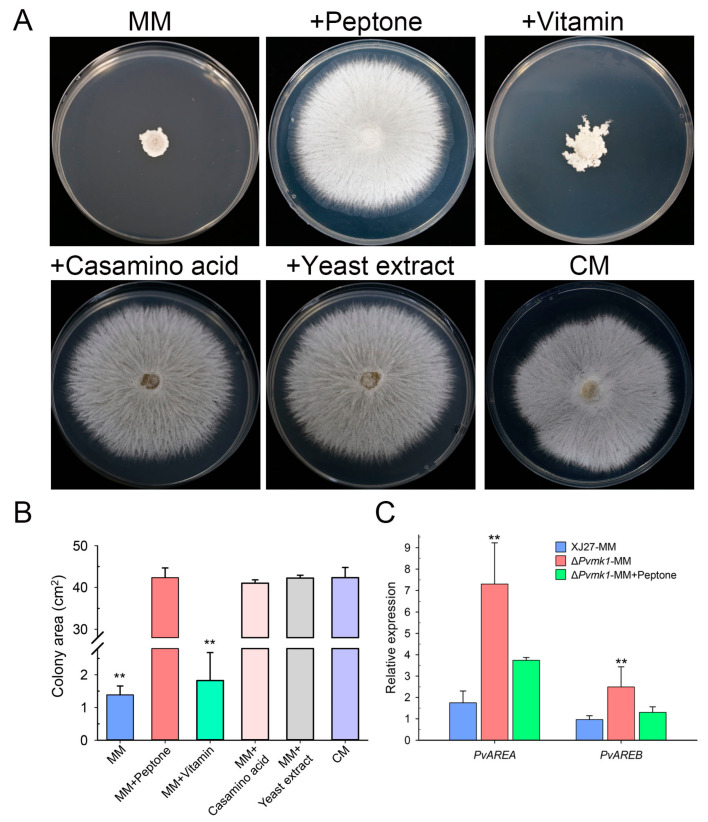
Test of nitrogen nutrient use by the Δ*Pvmk1* mutant. (**A**) Colony morphology of the mutant Δ*Pvmk1* grown on solid MM plates supplemented with different nutrient components for 6 d. (**B**) The colony area of the mutant Δ*Pvmk1* on different media. (**C**) The relative expression of nitrogen metabolism regulator genes *PvAREA* and *PvAREB* identified in the *P. versicolor* genome. The expression level of the *P. versicolor* β-tubulin gene was used as reference. Bars represent means and standard deviations calculated from three replications. Asterisks are used to indicate statistically significant differences (** *p* < 0.01).

The above observation that Δ*Pvmk1* mutants failed to grow on MM medium when compared to the colony diameter of the wild-type strain on the complete medium (CM) prompted us to hypothesize that the mutants might be defective in their tolerance to the relative nutrient-depleting conditions and to some of the key nutrient elements in the CM, but not in MM, that might be essential for the Δ*Pvmk1* mutant growth. To test this idea, we compared the nutrient elements of CM and MM and found that peptone, casamino acid, yeast extract, and amino acid solution from CM are absent in MM. We therefore added these elements into MM independently and tested the growth of the Δ*Pvmk1* mutants on these supplemented MM plates. Interestingly, we found that MM plates added with peptone, yeast extract, or casamino acid all restored the growth of Δ*Pvmk1* mutants to the level on CM plates, whereas the addition of amino acid solution only partially increased the growth of Δ*Pvmk1* mutants (Figure 3A,B). Since peptone and yeast extract are the main type of nitrogen nutrient in CM plates, these results suggest that the Δ*Pvmk1* mutants cannot grow normally on the MM medium without a sufficient nitrogen nutrient supply. Thus, the Δ*Pvmk1* mutants might be defective regardingtheir tolerance to nitrogen-starved stress compared to the wild-type strain, which can grow normally on MM even when the colony diameter is reduced relative to its growth on CM. Taken together, we conclude that PvMk1 is necessary for *P. versicolor* to tolerate and grow under nitrogen-starved conditions.

To further dissect how PvMk1 regulates nitrogen utilization, we analyzed the expression levels of two key transcription factors, PvAreA and PvAreB, the homologs of which are well-known negative regulators involved in fungal nitrogen metabolism (Gene sequences and primers used for qRT-PCR are listed in Supplementary Tables S1 and S2) [59,60]. The results showed that the transcript levels of both *PvAREA* and *PvAREB* genes were significantly increased in the Δ*Pvmk1* mutants compared to the wild-type strain, suggesting that nitrogen nutrient utilization might be highly inhibited in the Δ*Pvmk1* mutants due to the loss of PvMk1 (Figure 3C). Thus, we propose that PvMk1 is important for the regulation of nitrogen metabolism during *P. versicolor* growth.

### 3.5. PvMk1 Is Critical for P. versicolor Virulence on Bayberry

**Figure 4:**
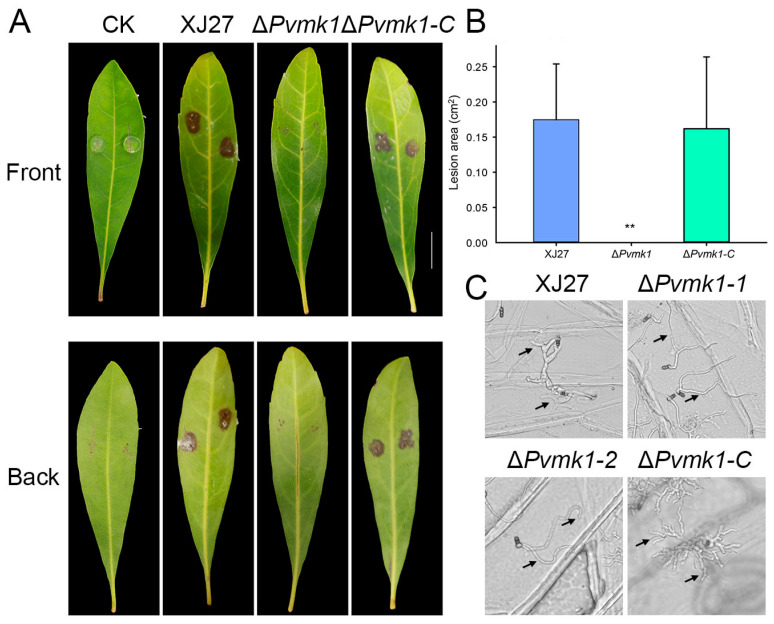
Virulence assays of the Δ*Pvmk1* mutant on bayberry leaves. (**A**) Leaves of Chinese bayberry (Cultivar ‘Zaojia’) were inoculated with the wild-type XJ27, the Δ*Pvmk1* mutant and the complemented strain Δ*Pvmk1-C*. Photographs of disease symptoms were taken 3 d after inoculation. (**B**) Calculation of lesion areas on Chinese bayberry leaves caused by different strains. Lesion area data were analyzed using Image J. Asterisks indicate statistically significant differences at ** *p* = 0.01 according to Duncan’s range test. Bar = 1 cm. (**C**) Conidia germination and infection structure differentiation of the XJ27, Δ*Pvmk1*, and Δ*Pvmk1-C* strains incubated on onion epidermal cells for 24 h. The arrow represents the point of invasion. Bars = 50 μm.

To explore the role of PvMk1 in the virulence of *P. versicolor* on bayberry, we inoculated the leaves of bayberry (*Myrica rubra*) with mycelial plugs from the wild-type strain XJ27, the Δ*Pvmk1* mutant, and the complemented strain Δ*Pvmk1-C*. Three days after inoculation, we observed that the wild-type XJ27 and the complemented strain Δ*Pvmk1-C* caused typical lesions on bayberry leaves. In contrast, the Δ*Pvmk1* mutant failed to cause any disease lesions, suggesting that the Δ*Pvmk1* mutant had lost virulence on the bayberry (Figure 4A,B).

Penetrating the epidermis is generally the first step involved in plant pathogenic fungi successfully infecting plants [61]. Since the mutant could not cause any disease spots on the leaves of *Myrica rubra*, we speculate that the mutant may have defects regarding the penetratation of the plant epidermis. To test this hypothesis, we observed the infection of the mutant on the onion epidermis. As shown in Figure 4C, after inoculating the onion epidermis for 24 h, the wild-type strain thickened its hyphae at the junction of the onion epidermal cells and expanded its tip, possibly representing the infection structure of *P. versicolor*, with some penetrating into the onion epidermis. In contrast, the mutant hyphae did not appear thickened or apically enlarged on the onion epidermis, nor did they penetrate the onion epidermis. This result indicates that the mutant has serious defects regaridng its ability to pentrate the plant epidermis. Taken together, we conclude that PvMk1 is critical for *P. versicolor* virulence on bayberry, and that it is possibly involved in regulating the formation of the infection structure that is required for plant surface penetration.

### 3.6. PvMk1 Is Involved in P. versicolor Responses to Environmental Stress Conditions

**Figure 5:**
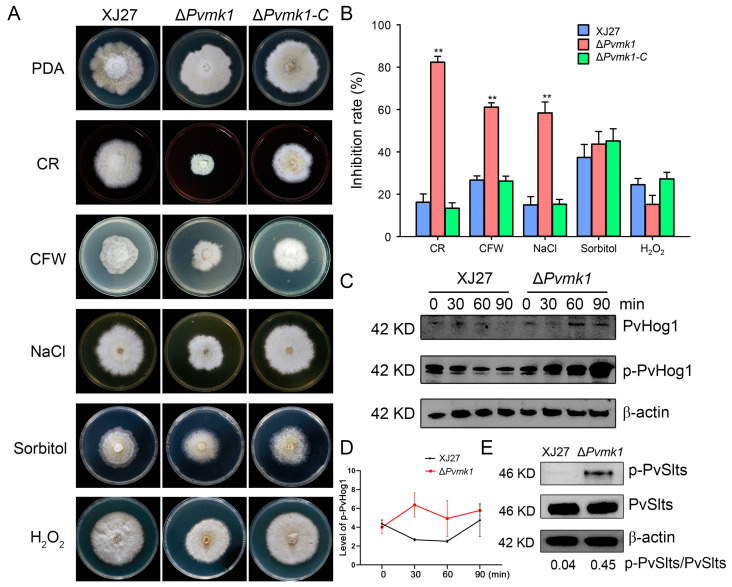
Assays of the response of the Δ*Pvmk1* mutant to cell wall integrity, hyperosmotic and oxidative stresses. (**A**) Colony morphology of different strains inoculated on PDA plates supplemented with the cell-wall-perturbing agent CR (100 μg/mL), CFW (300 μg/mL), osmotic stress agent NaCl (0.5 M), Sorbitol (2 M), and the oxidative stress agent H_2_O_2_ (20 mM). Colony photographs were taken at 6 DPI (days post-inoculation). (**B**) The growth inhibition rate of the XJ27, Δ*Pvmk1*, and Δ*Pvmk1-C* strains was calculated relative to their growth rate with untreated conditions. The data were analyzed using a GraphPad Prism 7.0 and an unpaired two-tailed Student’s t-test. Asterisks indicate statistically significant differences at ** *p* = 0.01. All experiments were repeated three times. (**C**) Assays of the phosphorylation level of PvHog1 in XJ27 and the Δ*Pvmk1* mutant after they were induced were performed by adding 0.5 M of NaCl at 0, 30, 60, and 90 min. The control was the protein content of β-actin. (**D**) The relative phosphorylated level of PvHog1 in XJ27 and the Δ*Pvmk1* mutant. Values were the ratio of phosphorylated PvHog1 to non-phosphorylated PvHog1 in indicated strains and time points. (**E**) Determination of the phosphorylation level of PvSlts (PvSlt1 or PvSlt2) in XJ27 and the Δ*Pvmk1* mutant cultured in CM with CFW stress treatment for 30 min.

To elucidate whether PvMk1 is involved in *P. versicolor* tolerance to exogenous stress conditions, XJ27, Δ*Pvmk1*, and Δ*Pvmk1-C* were inoculated on solid PDA plates supplemented with different stress drugs, including the cell-wall-damaging agents calcofluor white (CFW, 300 μg/mL) and Congo red (CR, 100 μg/mL) in order to test the cell wall integrity, 0.5 M of NaCl and 2 M of Sorbitol, which induce hypertonic stress, and 20 mM of H_2_O_2_, for oxidative stress analysis. After 5 d of culture, while the wild-type and the complemented strains showed no significant differences in their sensitivity to these stress drugs, the Δ*Pvmk1* mutant exhibited higher sensitivity to CR, CFW, and NaCl, suggesting that the Δ*Pvmk1* mutant was defective regarding its cell wall integrity and hypertonic tolerance, and had an especially high sensitivity to sodium. In contrast, there is no significant difference between the inhibition rate of the wild-type strain and the Δ*Pvmk1* mutant when treated with 20 mM of H_2_O_2_, suggesting that PvMk1 might not be involved in regulating the response of *P. versicolor* to oxidative stress (Figure 5A,B).

The stress response to external stress in fungi is usually controlled by MAPK signaling pathways [15,62]. To further explore the mechanisms of PvMk1 in regulating *P. versicolor* tolerance to stress conditions, we examined the phosphorylation level of PvSlt1 and PvSlt2, two *P. versicolor* homologs of the *S. cerevisiae* Slt2 MAP kinase responsible for cell wall integrity regulation, and PvHog1, a *P. versicolor* homolog of the yeast Hog1 kinase involved in the osmoregulation pathway [63,64]. The wild-type and mutant strains were cultured in CM and subjected to CFW treatment for 30 min. The total protein was extracted, and the phosphorylation level of the PvSlt1 and PvSlt2 proteins was detected using an anti-phospho-p44/42 MAPK antibody. The results showed that the phosphorylation level of the PvSlt1 and PvSlt2 proteins in the mutant was significantly higher than that of the wild type, further proving that the mutant had defects in its cell wall integrity, and the deletion of the PvMk1 gene damaged the cell wall integrity of *P. versicolor* (Figure 5E). In order to test the phosphorylation level of the PvHog1 protein, the wild type and mutant were sampled at different times after NaCl treatment, and the total protein was extracted. Using an anti-MAPK14(T180/Y182) antibody, the phosphorylation level of the PvHog1 protein was detected. The results show that, after treatment with 0.5 M of NaCl for 30 min, the PvHog1 phosphorylation level in XJ27 decreased over time. In contrast, the PvHog1 phosphorylation level in the Δ*Pvmk1* mutant increased higher and higher from 30 min to 90 min post-treatment. Moreover, the phosphorylation levels of PvHog1 in the Δ*Pvmk1* mutant were always higher than the wild-type strain XJ27, suggesting that PvMk1 is involved in controlling the PvHog1 phosphorylation level in response to hyperosmotic stresses (Figure 5C,D).

### 3.7. PvMk1 Is Required for P. versicolor Autophagy under Nutrient-Depleting Conditions

**Figure 6:**
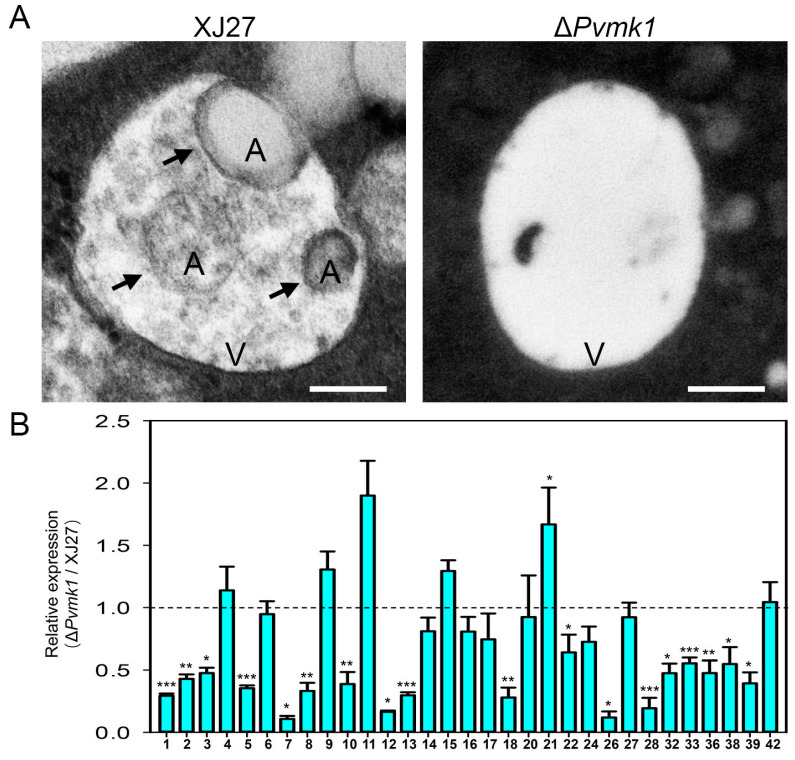
Assay of autophagosome formation and autophagy gene expression in the Δ*Pvmk1* mutant. (**A**) Observation of autophagosome formation in XJ27 and the Δ*Pvmk1* mutant using transmission electron microscopy (TEM). V represents vacuole, A represents autophagosome, indicated by arrows. Bar = 0.5 μm. (**B**) Global expression changes in 31 autophagy genes (ATGs) encoded by the *P. versicolor* genome. The expression level of *P. versicolor* β-tubulin gene was used to normalize different samples. The relative expression levels of each ATG gene in XJ27 was set as level 1. Bars represent means and standard deviations of gene expression levels in the Δ*Pvmk1* mutant compared to those in the wild-type XJ27 strain. All assays were performed in three replications. Significant differences between replicates were statistically evaluated using SDs and one-way ANOVA in SPSS. Asterisks indicate statistical differences between the wild type and the Δ*Pvmk1* mutant (* *p* < 0.05, ** *p* < 0.01, *** *p* < 0.001).

Cell autophagy is a conservative mechanism for the degradation and recycling of intracellular substances in eukaryotes in response to exogenous nutritional stress [34]. In this study, we found that the deletion of the *PvMK1* gene led to the inability of *P. versicolor* to grow on MM medium with relatively deficient nutrition. We speculate that the deletion of the *PvMK1* gene may affect the autophagy process of *P. versicolor*. To test this idea, we used transmission electron microscopy to observe the formation of lysosomes and internal autophagic bodies in the mycelium of the wild-type strain XJ27 and the Δ*Pvmk1* mutant induced by MM medium. The results showed that typical autophagic bodies could be observed in the vacuoles of the wild-type XJ27 strain, while in the mycelium of the Δ*Pvmk1* mutant, there were basically no autophagic bodies inside the lysosome (Figure 6A). These results suggest that PvMk1 regulates the autophagy process of *P. versicolor* under nutrient-deficient conditions.

Considering that PvMk1 is the most downstream protein kinase of the MAPK signaling pathway, which is responsible for activating or inhibiting the transcription and expression of whole cell genes, and that we have now confirmed that PvMk1 is necessary for the autophagy of *P. versicolor* cells, we would like to determine the regulatary role of PvMk1 in autophagy-related genes. According to the 42 autophagy-related genes (ATGs) identified in the model organism *S. cerevisiae*, and the relatively well-studied 32 ATGs in the model pathogenic fungus *M. oryzae*, we have retrieved 32 autophagy-related genes from the *P. versicolor* genome (gene sequences of 32 *PvATGs* are listed in Supplementary Table S2) [33,36]. In order to detect the expression of these genes during the autophagy process of *P. versicolor*, we extracted RNA from the wild-type and mutant strains cultured in MM, respectively, and reverse transcribed them into cDNA. qRT-PCR was used to detect the expression of these genes (primers used for qRT-PCR are listed in Supplementary Table S1). The results showed that 32 autophagy-related genes in *P. versicolor* were expressed to varying degrees in the wild type, while the expression level of 18 of 32 genes in the Δ*Pvmk1* mutant, including *PvATG1*, *PvATG2*, *PvATG3*, *PvATG5*, *PvATG7*, *PvATG8*, *PvATG10*, *PvATG12*, *PvATG13*, *PvATG18*, *PvATG22*, *PvATG26*, *PvATG28*, *PvATG32*, *PvATG33*, *PvATG36*, *PvATG38*, and *PvATG39*, was significantly decreased. In contrast, the expression level of *PvATG11* and *PvATG21* genes in the Δ*Pvmk1* mutant increased (Figure 6B). These results indicate that PvMk1 participates in the global regulation of the transcription and expression of autophagy-related genes in *P. versicolor*.

## 4. Discussion

Bayberry twig blight (BTB) is a devastating disease that has erupted on the *Myrica rubra* tree in recent years, causing the branches of *M. rubra* that are more than ten years of age to wither and the tree to finally die, leading to major losses in the bayberry industry. Understanding the pathogenic mechanism of the causal pathogen is important for formulating prevention and control strategies in production. Here, we isolated a *P. versicolor* pathogen from *M. rubra*, sequenced the genome of this fungus, and identified a MAPK kinase PvMk1 that controls the virulence of the strain in the genome using functional genomics. Our results showed that PvMk1 regulated the aerial hyphal development, conidia production, cell wall integrity, hypertonic stress, melanin synthesis and response to nitrogen nutrition stress of *P. versicolor*. We speculate that PvMk1 may play a key role in the pathogenesis of *M. rubra* by regulating these biological processes. Given that the pathogenic gene of *P. versicolor* has rarely been reported to date, this study provides a good starting point for further understanding the underlying pathogenic mechanisms of *P. versicolor* fungus and their effects on bayberry.

The PvMk1 protein of *P. versicolor* is a homolog of the yeast Fus3/Kss1 MAP kinases, which act as the central kinase in the signaling cascade responsible for the mating pheromone response and filamentation regulation [65]. We selected this gene for functional investigation due to its well-conserved role in fungal pathogenesis, as has been characterized in more than 20 fungal plant pathogens [15]. Thus, it is of interest to know whether this conserved Fus3/Kss1-type MAP kinase also participates in the virulence regulation of *Pestalotiopsis* pathogens. Our results demonstrated that, similar to previous studies, the deletion of *PvMK1* in the *P. versicolor* fungus nearly abolished its ability to cause disease on bayberry, suggesting the conserved role of this Fus3/Kss1-type MAP kinase PvMk1 in controlling the fungal pathogenesis of *Pestalotiopsis* pathogens. Besides virulence control, PvMk1 also regulates aerial hyphal development and the conidiation of *P. versicolor*. This is consistent with the role of its yeast homolog Fus3/Kss1, which is required for filamentous growth regulation.

The formation of conidia is an important reason for the spread and outbreak of fungal diseases (66). The pathogen of *M. rubra* wilt mainly infects the branches and leaves of *Myrica rubra* trees, and the conidia produced at the infected sites are the main means of transmitting *P. versicolor*. Therefore, understanding the molecular mechanism involved in the formation of conidia in *P. versicolor* is of great significance for managing disease transmission. However, there are few studies on the mechanism of conidia formation in *P. versicolor*. In this study, we found that after the deletion of the *PvMK1* gene, the ability of the mutant to sporulate decreased significantly. Compared to the wild type, which produces a large number of conidia in CM medium, the mutant is unable to produce conidia, indicating that PvMk1 is a key factor regulating the formation of conidia in *P. versicolor*. The formation of fungal conidia is generally located at the top of the hyphae, and the normal development of aerial hyphae is a prerequisite for the formation of conidia [66]. Therefore, we speculate that the inability of the Δ*Pvmk1* mutant to produce conidia may be due to the defective development of aerial hyphae. Indeed, our results indicate that when cultured on PDA, CM, and MM plates, the Δ*Pvmk1* mutant is unable to form aerial hyphae, confirming our hypothesis that the inability of PvMk1 to produce conidia is due to developmental defects in the aerial hyphae. The function of PvMk1 in regulating the development of aerial hyphae is a relatively conservative mechanism of Fus3/Kss1-type MAP kinase in fungi, as Fus3/Kss1 is primarily responsible for regulating the development of filamentous hyphae in yeast. However, further research is needed on how PvMk1 regulates the development of aerial hyphae in *P. versicolor*. A study on the development of airborne hyphae in *F. graminearum* has shown that autophagy-dependent nutrient supply is the key to the formation of airborne hyphae in *F. graminearum* [37]. This mechanism can also be used to explain why the *PvMK1* mutant cannot form aerial hyphae, as we have found that the cellular autophagy process of the Δ*Pvmk1* mutant is severely flawed and may not provide sufficient nutrients to ensure the development of the aerial hyphae.

We speculate that PvMk1 may regulate the formation of conidia in *P. versicolor* by regulating the activity of a downstream transcription factor. Previous studies have shown that Mcm1, as a transcription factor downstream of Pmk1, regulates the formation of conidia. The deletion of the MoMCM1 gene resulted in a 10-fold decrease in the yield of conidia in the mutant compared to the wild type [67]. There is a homologous protein of MoMcm1 in *P. versicolor*, PvMcm1, that has a typical transcription factor domain (data not shown). The next step is to investigate whether PvMcm1 regulates the spore production of *P. versicolor*, and the interaction between PvMcm1 and PvMk1, which will help to explain the molecular mechanism of PvMk1 and its regulation of the conidial formation of *P. versicolor*.

In addition to basic growth and development regulation, fungi also need to cope with various environmental conditions and adverse factors in order to survive better in nature. The regulatory mechanism of this process has been thoroughly elucidated in yeast, and is mediated by different MAPK signaling pathways [16]. Fus3/Kss1 MAP kinase is responsible for regulating pheromone sensing and hyphal development, Slt2 MAP kinase is responsible for regulating cell wall integrity stress, and the Hog1 signaling pathway is responsible for regulating high osmotic stress. Therefore, based on the homology between PvMk1 and the yeast Fus3/Kss1 MAP kinase, it is reasonable that PvMk1 should play a major role in regulating the mycelial development of *P. versicolor*. However, our results concerning the *PvMK1* mutants under different stress conditions showed that PvMk1 also participates in regulating the response of *P. versicolor* to cell wall damage and hyperosmotic stress. Similar results have been reported in other plant pathogenic fungi. For example, in the anthracnose-causing fungi *Colletotrichum higginsianum* and *C. scovillei*, the deletion of the Fus3/Kss1-type MAPKs renders the mutants more sensitive to cell wall integrity inhibitors and osmotic stresses [68,69]. However, in contrast to our results, the deletion of CfMK1 in *C. fructicola* enhanced the tolerance of the mutants to cell wall and plasma membrane stresses, suggesting the distinctive roles of Fus3/Kss1-type MAPKs played in different fungi [70].

One open question is how PvMk1 simultaneously regulates the response of *P. versicolor* to multiple environmental stresses. What are the cross-talking mechanisms between PvMk1 and the Slt2 and Hog1 homologous MAPKs in *P. versicolor*? One possible explanation is that there might be some common components upstream of different MAPKs that help to transduce similar signals. Indeed, it has been previously established that the scaffold protein Ste50 that works upstream of the three-tied MAPK cascades is highly conserved and shared by different MAPK signaling pathways in filamentous fungi [71]. Its function mechanism has been well characterized in the rice blast fungus, in which the Ste50 homolog Mst50 protein directly interacts with the Mst11 kinase of the Pmk1 pathway responsible for the formation of the infection structure, the Bck1 and Ssk2 kinases in the Mps1 pathway responsible for the cell wall stress response, and the histidine kinase Hik1, which functions upstream of Osm1 and is involved in high-osmotic regulation [30,72]. A more recent study further demonstrated that a membrane-localized receptor, MoOpy2, directly interacts with Mst50 to sense and transduce external stimuli [50]. Thus, Mst50 functions as a signaling harbor in order to coordinate all the three MAPK pathways in *M. oryzae* via the transduction of external or plant signals. Considering the conservation of the Mst50 and MoOpy2 proteins in the *P. versicolor* genome (data not shown), we propose that this cross-talking mechanism of multiple MAPK signaling pathways might also be involved in the regulation of the *P. versicolor* response to various external signals during development and pathogenesis.

One remarkable discovery of our study is that the deletion of *PvMK1* renders the fungus unable to grow on MM medium, compared to the colony diameter of the wild-type strain on the CM medium. This observation indicates that MM lacks some of the nutrient components that can be directly utilized by the *PvMK1* mutant. In contrast, the wild-type strain harboring PvMk1 can cope with this relatively nutrient-deficient stress and maintain a certain degree of mycelial growth. Further research found that peptone, yeast extract, or casamino acid components were necessary for the growth of the Δ*Pvmk1* mutant, indicating the role of PvMk1 in regulating nitrogen utilization. A large number of studies have shown that the response of fungi to nitrogen-source nutritional stress usually involves autophagy, which involves the degradation of their own aging organelles or damaged proteins for nutrient cycling in order to cope with transient hunger stress [31,32,33,34,35,36]. Therefore, we speculate that the wild-type *P. versicolor* may have initiated a cellular autophagy mechanism in order to maintain mycelial growth on relatively nutrient-deficient MM media, while in the *PvMK1* mutants, this cellular autophagy mechanism is not effectively activated, resulting in the mutant being unable to grow due to nutrient deficiency. Our results confirmed this hypothesis. We observed a large number of lysosomal vesicles rich in autophagic bodies in wild-type strains, but this phenomenon was rare in the Δ*Pvmk1* mutants. The global differential expression of genome-wide ATG genes in the Δ*Pvmk1* mutant further confirms that PvMk1 plays an important and central role in the regulation of cell autophagy. These results are consistent with studies on the rice blast fungus *M. oryzae*, in which the deletion of the pathogenicity MAP kinase Pmk1 abolished fungal autophagy and plant infection. A recent study showed that Pmk1 controls *M. oryzae* autophagy by modulating the activity of the downstream transcription factor MoHox7 [42]. We speculate that PvMk1 may interact with the MoHox7 homolog in *P. versicolor*, and has thus adapted a mechanism similar to *M. oryzae* in order to regulate the autophagy process of *P. versicolor* under nutritional stress.

Autophagy has been demonstrated to be essential for the formation of the specialized infection structure appressorium in many plant pathogenic fungi [35,36,41] Although a typical spherical appressorium structure has not been observed during *P. versicolor* infection, we found that the *P. versicolor* hyphae become thickened and swollen on the onion epidermis, similar to the infection structure hyphopodium reported in the vascular wilt pathogen *V. dahliae* [73]. This indicates that *P. versicolor* can also produce a specifically differentiated infection structure when infecting plants, and that the formation of this infection structure is regulated by PvMk1.

In summary, this work was carried out to understand the molecular basis of the recently outbroken BTB disease caused by the *P. versicolor* fungus on *M. rubra* trees. As a point of starting research on the molecular mechanisms of *Pestalotiopsis* pathogenesis, we isolated a highly virulent *P. versicolor* strain, XJ27, sequenced the whole genome, and for the first time, used homologous recombination technology to knock out pathogenicity-related genes for functional characterization. This work has laid a good foundation for further investigations into the molecular interaction mechanisms between *P. versicolor* and *M. rubra*. Our findings have established that PvMk1 plays multiple roles in the pathogenesis of *P. versicolor* and is a key virulence determinator in BTB disease development. Future research focusing on the identification of the upstream kinase elements involved in the PvMk1 signaling pathway and cell membrane-localized signal sensing receptors, as well as the downstream transcription factors that are directly activated or inhibited by PvMk1, will help us to better understand the molecular mechanisms of *P. versicolor* infection and disease development.

## 5. Conclusions

In conclusion, our findings have revealed that PvMk1 is required for hyphal development, conidiation, stress response, nitrogen metabolism, and the autophagy of *P. versicolor*. Together, these regulatory functions might contribute to the critical role of PvMk1 as a virulence determinator in *P. versicolor* causing bayberry twig blight diseases.

## Figures and Tables

**Table 1:** Statistics of the *P. versicolor* XJ27 genome sequencing and annotation.

Variables	Statistics
Genome size (Mb)	50.74
Number of scaffolds	8
Scaffold N50 size (Mb)	7.26
GC content (%)	50.14%
Number of predicted genes	16455
Average gene length (bp)	665
Number of predicted secreted proteins	1884
BUSCO ^1^ completeness (%)	99.8%

^1^ BUSCO = Benchmarking Universal Single-Copy Ortholog.

## Data Availability

The data are contained within the article or in the Supplementary Materials.

## Supplementary Materials

The following supporting information can be downloaded at: https://www.mdpi.com/article/10.3390/jof9060606/s1, Figure S1: *PvMK1* gene knockout and identification of deletion mutants. Table S1: Primers used in this study. Table S2: Gene sequences of *P. versicolor* used for qRT-PCR analysis.
