# Supplementary material for: The MAP Kinase PvMK1 Regulates Hyphal Development, Autophagy, and Pathogenesis in the Bayberry Twig Blight Fungus Pestalotiopsis versicolor
Source: J Fungi (Basel). 2023 May 24;9(6):606. doi: 10.3390/jof9060606 (PMC10299636; doi:10.3390/jof9060606)
Supplement: Supplementary file 1 [file jof-09-00606-s001.zip › Figure S1.pdf]

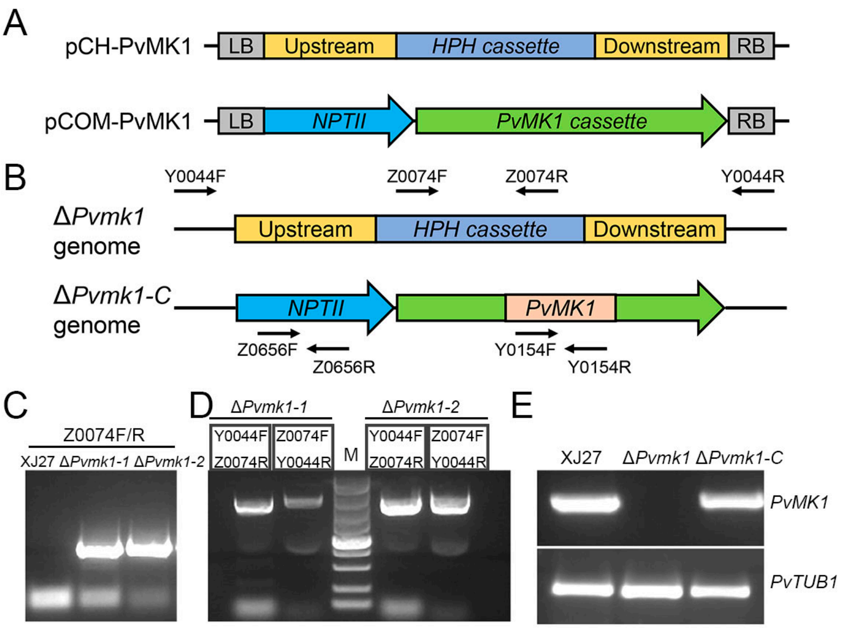

Figure S1. PvMK1 gene knockout and identification of deletion mutant. (A) Physical map of PvMK1 gene replacement vector pCH-PvMK1 and complementation vector pCOM-PvMK1. (B) Schematic representation of PCR primers for confirmation of gene deletion and complementation. (C) Confirmation of HPH genes in two transformants ΔPvmk1-1 and ΔPvmk1-2. The wild type XJ27 was used as a control. (D) Confirmation of PvMK1 gene deletion by combined primers shown in (B), of which primers Y0044F and Y0044R were located near the gene replacement region to confirm targeted gene deletion, but not random insertion. (E) RT-PCR analysis of PvMK1 gene expression in the complemented strain ΔPvmk1-C, compared with its absence in the deletion mutant ΔPvmk1.
